# Dermal Olfactory Receptor OR51B5 Is Essential for Survival and Collagen Synthesis in Human Dermal Fibroblast (Hs68 Cells)

**DOI:** 10.3390/ijms22179273

**Published:** 2021-08-27

**Authors:** Bomin Son, Wesuk Kang, Soyoon Park, Dabin Choi, Taesun Park

**Affiliations:** Department of Food and Nutrition, BK21 FOUR, Yonsei University, 50 Yonsei-ro, Seodaemun-gu, Seoul 120-749, Korea; mim1110@naver.com (B.S.); wesuk42@naver.com (W.K.); thdbs1201@naver.com (S.P.); vin1411@naver.com (D.C.)

**Keywords:** OR51B5, collagen, cell survival, dermal fibroblasts

## Abstract

Skin dermis comprises extracellular matrix components, mainly collagen fibers. A decrease in collagen synthesis caused by several factors, including ultraviolet (UV) irradiation and stress, eventually causes extrinsic skin aging. Olfactory receptors (ORs) were initially considered to be specifically expressed in nasal tissue, but several ORs have been reported to be present in other tissues, and their biological roles have recently received increasing attention. In this study, we aimed to characterize the role of ORs in cell survival and collagen synthesis in dermal fibroblasts. We confirmed that UVB irradiation and dexamethasone exposure significantly decreased cell survival and collagen synthesis in Hs68 dermal fibroblasts. Moreover, we demonstrated that the mRNA expression of 10 ORs detectable in Hs68 cells was significantly downregulated in aged conditions compared with that in normal conditions. Thereafter, by individual knockdown of the 10 candidate ORs, we identified that only *OR51B5* knockdown leads to a reduction of cell survival and collagen synthesis. *OR51B5* knockdown decreased cAMP levels and dampened the downstream protein kinase A/cAMP-response element binding protein pathway, downregulating the survival- and collagen synthesis-related genes in the dermal fibroblasts. Therefore, OR51B5 may be an interesting candidate that plays a role in cell survival and collagen synthesis.

## 1. Introduction

The dermis mainly comprises extracellular matrix (ECM) components, including collagens, which account for 90% dry weight of the skin. Dermal collagen is synthesized by dermal fibroblasts and is responsible for the tensile strength and mechanical characteristics of the skin. However, reduced collagen synthesis due to intrinsic and extrinsic factors results in skin aging, including wrinkle formation, sagging, and laxity [1,2]. Furthermore, the disorganization of the dermal ECM has significant repercussions well beyond cosmetic alterations to the skin, and it has been suggested that aging locally occurring in the dermal ECM contributes to systemic aging [3,4], which redefines the perspective on skin aging.

Skin aging is the combined consequence of genetically programmed processes (intrinsic aging) and environmental damage (extrinsic aging). Notably, it is known that the majority of skin aging is mediated by extrinsic factors [5,6,7]; the treatment of extrinsic aging is the focus of much attention [8,9,10]. Numerous studies have indicated that at least two factors are primarily involved in extrinsic aging, namely (1) ultraviolet (UV) radiation and (2) stress. It has been clearly demonstrated that these stimuli cause a reduction in cell number and collagen synthesis in dermal fibroblasts [11,12,13,14,15], but the mechanisms involved in the regulation of cell growth and collagen synthesis are not fully established.

Olfactory receptors (ORs) constitute the largest G-protein-coupled receptor family and were originally assumed to be expressed exclusively in the olfactory tissue. Nonetheless, it has recently been found that ORs are much more versatile than initially thought; they are emerging as general chemoreceptors found in diverse tissues (e.g., skin, colon, liver, fat, and muscle) and their expression is related to numerous pathological processes [16,17,18,19]. For example, OR family 10 subfamily A member 7 (*OR10A7*) gene expression was significantly upregulated in the epidermal tissues of atopic dermatitis patients compared with healthy subjects; the knockdown of this receptor attenuated the allergen-induced inflammatory response in keratinocytes [20]. In addition, it has been reported that *OR7C1* mRNA is preferentially expressed in colon cancer cells than in normal colon cells; *OR7C1* knockdown delayed tumor progression in several colorectal cancer cell lines [21]. In the present study, we aimed to characterize the changes in the OR expression profile in dermal fibroblasts exposed to UV light and stress, which are well-known to reduce cell survival and collagen synthesis. We further sought to explore the roles of specific ORs involved in dermal fibroblast aging by individual candidate OR knockdown and their molecular mechanisms.

## 2. Results

### 2.1. Inhibitory Effects of UVB or Dexamethasone on Hs68 Cell Viability

**Figure 1:**
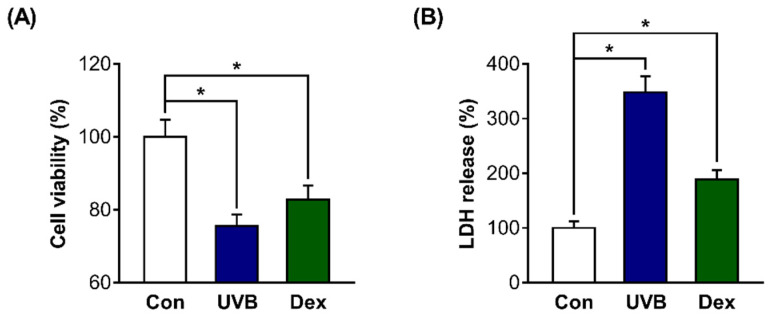
Inhibitory effects of ultraviolet B (UVB) radiation or dexamethasone (Dex) on Hs68 cell survival. (**A**) The cells were treated with 20 mJ/cm^2^ UVB or 1 μM Dex, or without them (control (Con)). After treatment for 24 h, the cell viability was determined using a water-soluble tetrazolium salt-1 (WST-1) assay. (**B**) The death of the cells treated with UVB or Dex was analyzed by lactate dehydrogenase (LDH) assay. The results are expressed as mean ± standard error of mean (SEM) of three independent experiments. * *p* < 0.05.

On the basis of previous studies [22,23,24,25,26], we first attempted to confirm whether UV irradiation or dexamethasone (Dex) treatment efficiently works in dermal fibroblasts in the present study. We confirmed that the mRNA expression of p16 and β-galactosidase activity (well-known cellular senescence markers) were significantly increased after treatment with 20 mJ/cm^2^ UVB or 1 µM Dex (Figure S1). Then, we aimed to use UVB and Dex to cause the reduction of survival in Hs68 cells. We measured cell viability after treatment with UVB or Dex, and confirmed that the viability was significantly decreased in UVB-irradiated or Dex-treated Hs68 cells compared with untreated control cells (Con). The absolute values of absorbance for the cell viability from the CON, UVB, and Dex groups were 1.074, 0.811, and 0.890, respectively (Figure 1A). In addition, the release of lactate dehydrogenase (LDH) was significantly increased in UVB-irradiated or Dex-treated Hs68 cells, indicating that reduced cell viability by UVB or Dex is associated with cell death (Figure 1B).

### 2.2. Inhibitory Effects of UVB or Dex on Collagen Synthesis in Hs68 Cells

**Figure 2:**
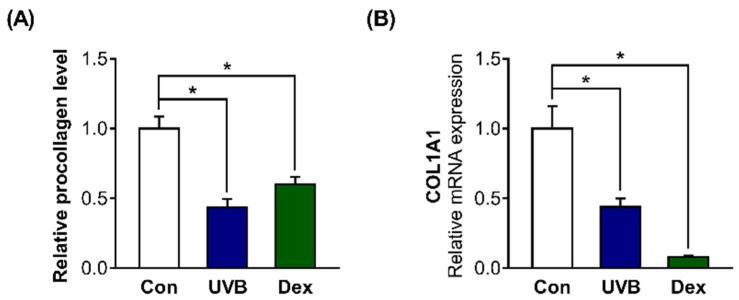
Inhibitory effects of UVB radiation or Dex on collagen synthesis in Hs68 cells. The cells were treated with or without 20 mJ/cm^2^ UVB or 1 μM Dex. (**A**) After treatment for 24 h, the relative amount of type-1 procollagen was measured in the culture supernatants using enzyme-linked immunosorbent assay (ELISA). The amount of procollagen in each sample was normalized to the total cellular protein content. (**B**) After UVB or Dex treatment for 12 h, collagen type 1 alpha 1 chain (*COL1A1*) mRNA levels were analyzed by quantitative polymerase chain reaction (PCR). Glyceraldehyde 3-phosphate dehydrogenase (GAPDH) was used as the reference gene for PCR. The results are expressed as mean ± SEM of three independent experiments. * *p* < 0.05.

We further examined whether these factors affect collagen synthesis in Hs68 cells. The relative procollagen content in the cell culture medium was significantly decreased by UVB or Dex treatment (Figure 2A). We also found that collagen type 1 alpha 1 chain (*COL1A1*) mRNA levels were greatly decreased in UVB-irradiated or Dex-treated Hs68 cells, which is consistent with the decreased collagen content (Figure 2B).

### 2.3. Altered Patterns of OR Gene Expression in UVB-Irradiated or Dexamethasone-Induced Hs68 Cells

**Figure 3:**
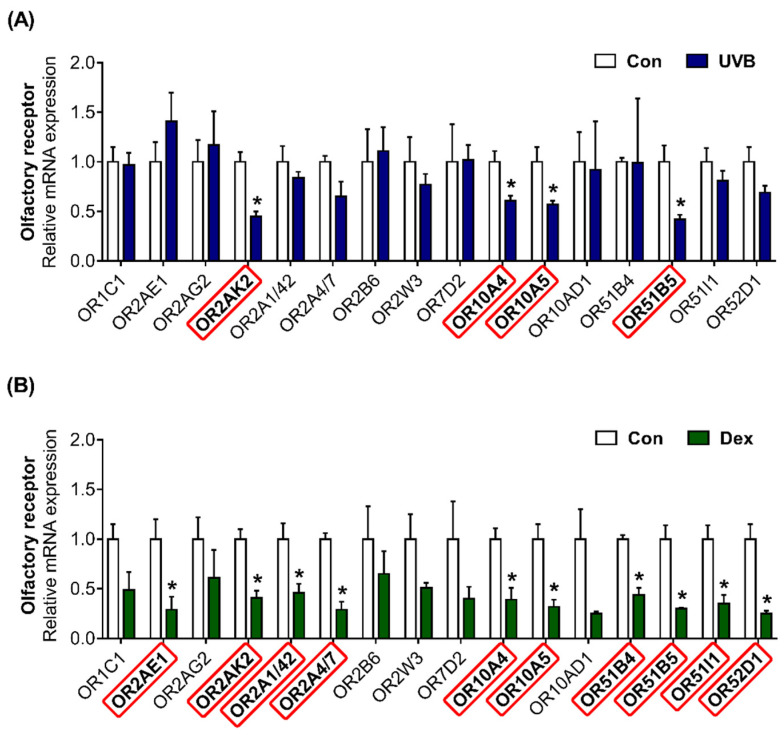
Altered patterns of olfactory receptor (OR) gene expression in UVB-irradiated or Dex-induced Hs68 cells. The cells were treated with or without (**A**) 20 mJ/cm^2^ UVB or (**B**) 1 μM Dex. After treatment for 12 h, the relative mRNA expression of 16 ORs (*OR1C1*, *OR2AE1*, *OR2AG2*, *OR2AK2*, *OR2A1/42*, *OR2A4/7*, *OR2B6*, *OR2W3*, *OR7D2*, *OR10A4*, *OR10A5*, *OR10AD1*, *OR51B4*, *OR51B5*, *OR51I1*, and *OR52D1*) were analyzed. GAPDH was used as the reference gene for PCR. Red rectangle represents differentially expressed genes between each treatment and control group (Con). The results are expressed as mean ± SEM of three independent experiments. * *p* < 0.05.

Using next-generation sequencing (NGS) data, we previously found that 16 ORs are highly expressed in Hs68 cells (NCBI Bioproject PRJNA736226; BioSample accession number SAMN 19613594). We confirmed that olfactory signaling pathway components, including ric8b, adenylate cyclase type 3 (ADCY3), and G protein subunit α L (GNAL), are present in Hs68 cells (Figure S2). The OR gene expression was quantified to investigate the effects of UVB or Dex treatment in Hs68 dermal fibroblasts. In UVB-exposed cells, the transcriptional expression of OR2AK2, OR10A4, OR10A5, and OR51B5 was effectively decreased (Figure 3A). Dex treatment significantly decreased the gene expression of 10 ORs (OR2AE1, OR2AK2, OR2A1/42, OR2A4/7, OR10A4, OR10A5, OR51B4, OR51B5, OR51I1, and OR52D1; Figure 3B).

### 2.4. OR51B5 Knockdown Decreased Hs68 Cell Viability

**Figure 4:**
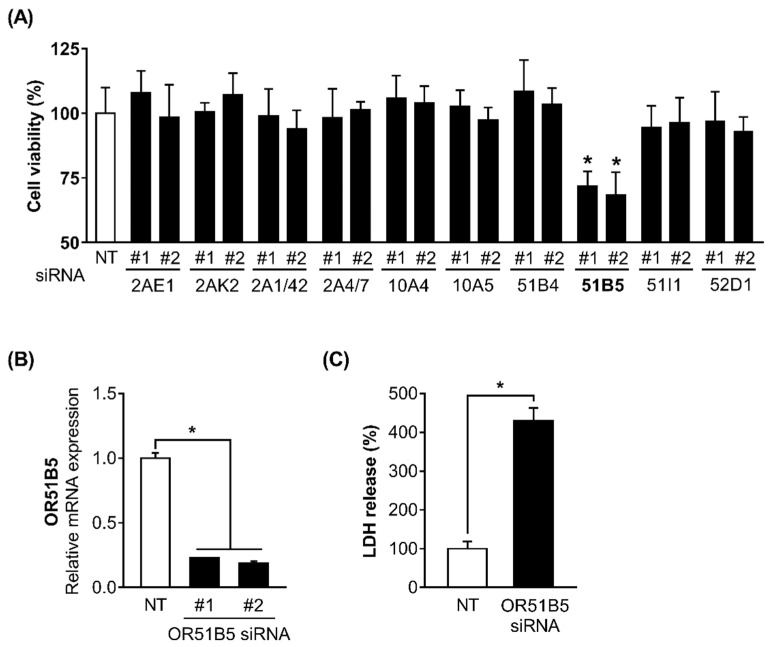
The knockdown of *OR51B5*, among the 10 altered OR genes, decreased Hs68 cell viability. (**A**) The cells were transfected with non-targeting small interfering RNA (siRNA; NT) or two distinct siRNAs (#1 and #2) against 10 ORs (*OR2AE1*, *OR2AK2*, *OR2A1/42*, *OR2A4/7*, *OR10A4*, *OR10A5*, *OR51B4*, *OR51B5*, *OR51I1*, and *OR52D1*) and further incubated for 72 h. Cell viability was analyzed using WST-1 assay. NT or *OR51B5*-specific siRNA #1 and #2 were introduced into Hs68 cells and cells were harvested after 72 h. (**B**) *OR51B5* mRNA expression and (**C**) cell death were analyzed by quantitative PCR and LDH assay, respectively. The results are expressed as mean ± SEM of three independent experiments. * *p* < 0.05.

We hypothesized that some of the 10 altered OR genes may play a role in regulating cell survival in Hs68 cells. To test this, we decided to use siRNA screening approach and measured the viability of the Hs68 cells transfected with non-targeting small interfering RNA (siRNA; NT) or two distinct siRNAs (#1 and #2) against 10 ORs (*OR2AE1*, *OR2AK2*, *OR2A1/42*, *OR2A4/7*, *OR10A4*, *OR10A5*, *OR51B4*, *OR51B5*, *OR51I1*, and *OR52D1*). Viability was significantly decreased only in *OR51B5* siRNA-transfected cells compared with NT-transfected cells (Figure 4A). To confirm the knockdown efficiency and select the more effective siRNA between the two siRNAs (siRNA #1 and siRNA #2) targeting different regions in the *OR51B5* mRNA, we measured the *OR51B5* expression in Hs68 cells transfected with NT or *OR51B5* siRNA #1 and #2. The *OR51B5* expression was decreased by 77.1 and 81.4% in *OR51B5* siRNA #1- or #2-transfected Hs68 cells, respectively (Figure 4B). Hence, *OR51B5* siRNA #2 was used to knockdown the *OR51B5* expression in the subsequent experiments. We further confirmed that the LDH was significantly elevated in *OR51B5* siRNA-transfected cells compared with NT-transfected cells, suggesting that reduced cell viability by *OR51B5* knockdown is related to cell death (Figure 4C).

### 2.5. OR51B5 Knockdown Decreased Collagen Synthesis in Hs68 Cells

**Figure 5:**
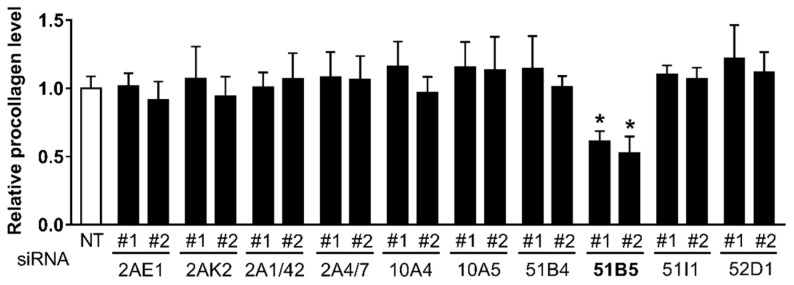
The knockdown of *OR51B5*, among the 10 altered OR genes, decreased the collagen synthesis in Hs68 cells. The cells were transfected with NT or two distinct siRNA #1 and #2 against 10 ORs, the same as for Figure 4, and further cultured for 72 h. Type-1 procollagen was determined in the culture supernatants using ELISA. The amount of procollagen in each sample was normalized to the total cellular protein content. The results are expressed as mean ± SEM of three independent experiments. * *p* < 0.05.

Likewise, to determine whether some of the 10 altered OR genes affected collagen synthesis in Hs68 cells, we also measured collagen synthesis in Hs68 cells transfected with NT siRNA or two distinct siRNAs #1 and #2 against 10 ORs. Consistent with the decreased cell viability, collagen synthesis was reduced only in *OR51B5* siRNA-transfected cells (Figure 5).

### 2.6. OR51B5 Knockdown Inhibited Olfactory Signaling Pathway in Hs68 Cells

**Figure 6:**
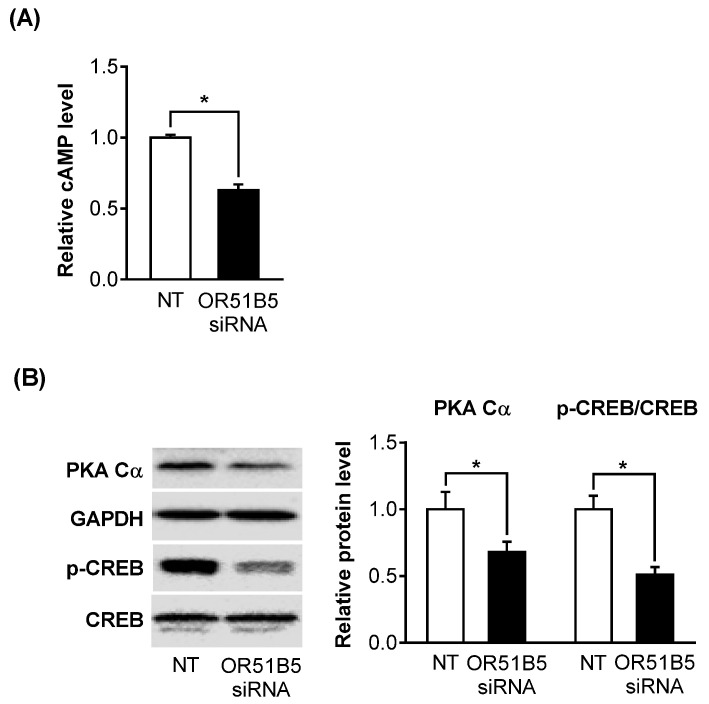
*OR51B5* knockdown inhibits the olfactory signaling pathway in Hs68 cells. (**A**) Intracellular cyclic adenosine monophosphate (cAMP) levels were determined by cAMP ELISA kit after transfection with non-targeting siRNA (NT) or *OR51B5*-specific siRNA #2. (**B**) Protein expression of protein kinase A catalytic subunit (PKA Cα), glyceraldehyde 3-phosphate dehydrogenase (GAPDH), phosphorylated cAMP-response element-binding protein (p-CREB), and CREB was determined by Western blot. Results are expressed as mean ± SEM of three independent experiments. * *p*  <  0.05.

As OR51B5 belongs to the G protein-coupled receptor superfamily, we hypothesized that the decreased *OR51B5* expression affects its downstream effectors, including cyclic adenosine monophosphate (cAMP), protein kinase A (PKA), and cAMP-response element binding protein (CREB). Indeed, *OR51B5* knockdown significantly decreased cAMP levels, PKA catalytic subunit (PKA Cα) protein expression, and CREB phosphorylation level in Hs68 cells (Figure 6A,B).

### 2.7. OR51B5 Knockdown Decreased Cell Survival-Related Gene Expression in Hs68 Cells

**Figure 7:**
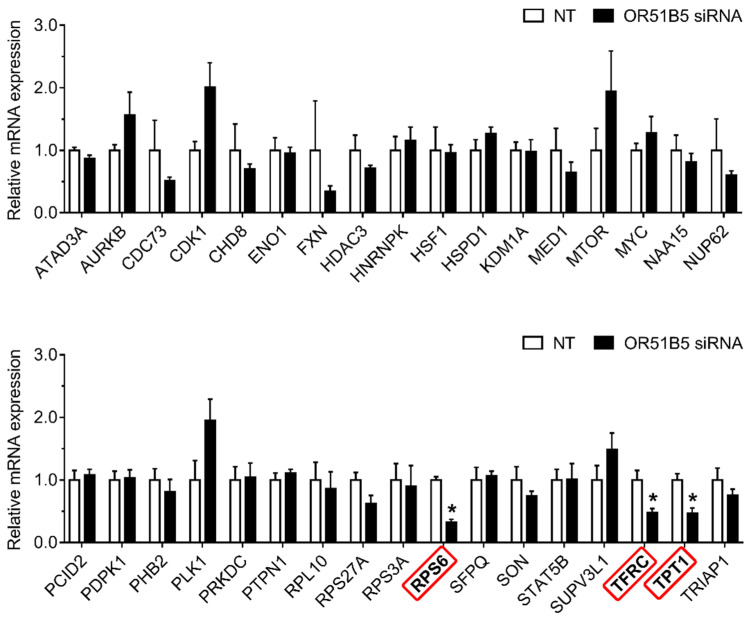
*OR51B5* knockdown decreased cell survival-related gene expression in Hs68 cells. The cells were transfected with siRNA targeting *OR51B5* or non-targeting siRNA (NT) and were further cultured for 72 h. The relative mRNA levels of cell survival-related genes were measured by quantitative PCR. Gene abbreviations are defined in supplementary Table S1. The results are expressed as mean ± SEM of three independent experiments. * *p*  <  0.05.

To investigate whether *OR51B5* knockdown-induced cell growth inhibition is associated with cell survival-related genes, we first selected 34 cell survival-related genes transcriptionally regulated by CREB [27,28,29]. Thereafter, we evaluated their expression in NT- or *OR51B5* siRNA #2-transfected Hs68 cells. Among these, the gene expression of three genes including ribosomal protein s6 (*RPS6*), transferrin receptor (*TFRC*), and tumor protein, translationally-controlled 1 (*TPT1*) was significantly lower after *OR51B5* siRNA transfection than that after NT transfection (Figure 7).

### 2.8. OR51B5 Knockdown Decreased Collagen Synthesis-Related Gene Expression in Hs68 Cells

**Figure 8:**
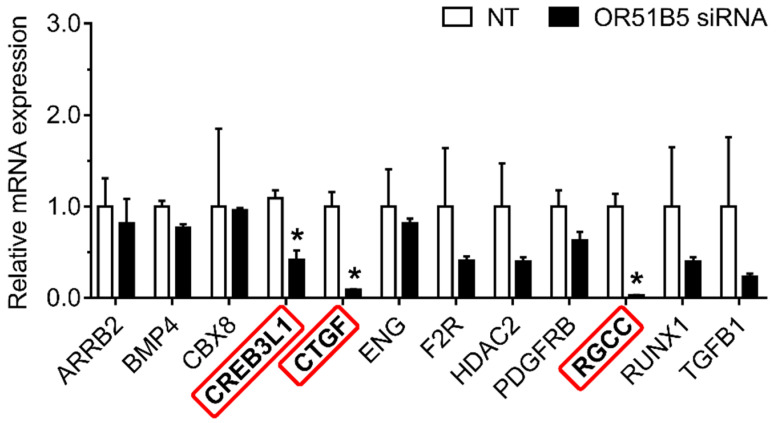
*OR51B5* knockdown decreased collagen synthesis-related gene expression in Hs68 cells. The cells were transfected with siRNA targeting *OR51B5* or non-targeting siRNA (NT) and were further incubated for 72 h. The relative mRNA levels of collagen synthesis-related genes were measured using quantitative PCR. Gene abbreviations are defined in supplementary Table S1. The results are expressed as the mean ± SEM of three independent experiments. * *p*  <  0.05.

**Figure 9:**
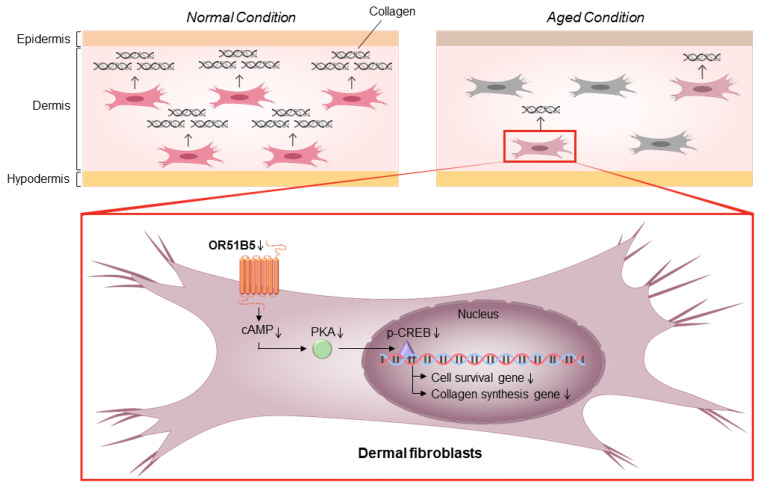
A proposed mechanism of OR51B5-mediated cell survival and collagen synthesis in Hs68 cells. Cell survival and collagen synthesis can be regulated through OR51B5-induced cAMP and the downstream PKA pathway. Then, PKA phosphorylates CREB protein, affecting cell survival- and collagen synthesis-related gene transcription. The thin downward arrows depict the downregulation of molecules related to the survival and collagen synthesis in OR51B5-knockdown cells. The bold arrows linking the molecules depict the signal transduction of these molecules.

To determine whether *OR51B5* knockdown-induced collagen synthesis inhibition is associated with collagen synthesis-related genes, we chose 12 collagen synthesis-related genes transcriptionally regulated by CREB [28,29]. We measured their expression in NT- or *OR51B5* siRNA #2-transfected Hs68 cells. Among these, the gene expression of three genes, including cAMP responsive element binding protein 3 like 1 (*CREB3L1*), connective tissue growth factor (*CTGF*), and regulator of cell cycle (*RGCC*), was significantly lower after *OR51B5* siRNA transfection than that after NT transfection (Figure 8). Taken together, it is likely that *OR51B5* knockdown decreases cell survival and collagen synthesis with the potential involvement of the cAMP/PKA/CREB pathway, downregulating the survival- and collagen synthesis-related genes in dermal fibroblasts (Figure 9).

## 3. Discussion

OR activation by odorants or other stimuli in the olfactory sensory neurons located in the nasal epithelium stimulates a specific olfactory G-protein (GNAL), which sequentially switches on ric8b and ADCY3 and causes cAMP production [30,31,32]. Although the produced cAMP is mainly associated with the transmission of odor signals to the olfactory system in the brain, a transient increase in intracellular cAMP also stimulates the downstream PKA–CREB pathway, which modulates various pathological and physiological functions [33,34,35,36]. Notably, olfactory signaling components are distributed not only in the olfactory cells; however, several studies, including our previous work, have demonstrated that several cell types in ectopic tissues constitutively express olfactory transduction machinery components, such as ORs, GNAL, ric8b, and ADCY3 [19,32,37,38]. Consistent with the results of previous studies, we confirmed that major olfactory signaling components, such as ORs, *GNAL*, *ric8b*, and *ADCY3*, are expressed in human dermal fibroblasts. Thus, it can be speculated that ectopically expressed OR activation may target the cAMP–PKA pathways, thereby mediating various functions, including cell survival and collagen synthesis.

As ORs have been found outside the olfactory epithelium, the biological functions of ectopically expressed ORs, including OR51B5, have been discovered in recent years, and previous studies have shown that the biological roles of OR51B5 are involved in the anti-leukemic effect in white blood cells, and the wound healing effect in epidermal keratinocytes [39,40]. In this study, we found that OR51B5 is significantly expressed in dermal fibroblasts and is related to cell survival and collagen biosynthesis. Our findings strongly support the view that ectopically expressed ORs in specific cells have a specialized function in that cell, irrespective of olfactory function. Notably, the Human Protein Atlas, a public database of protein expression profiles, consistently indicates that OR51B5 is specifically expressed in some human tissues, including skin, blood, reproductive organs, and kidneys [41]. Therefore, information on ORs distribution in the human body provides a clue to the unidentified functions of the specific OR. It would be intriguing to explore the additional roles of OR51B5 in reproductive tissues and kidneys in the future.

In this study, UVB irradiation or dexamethasone (a synthetic glucocorticoid) treatment were used for developing in vitro skin aging models representing UV-irradiated and stressful conditions. UV is classified into three wavebands: UVA (315–400 nm), UVB (280–315 nm), and UVC (100–280 nm). Among them, UVC is selectively blocked by the ozone layer. In addition, although sunlight contains approximately 20 times more UVA than UVB, it is widely accepted that UVB is the leading cause of UV-irradiated skin damage owing to its high energy and direct interaction with DNA to synthesize harmful photoproducts, including pyrimidine–pyrimidine complexes [42,43,44,45]. On the other hand, physiological stress triggers corticotropin-releasing factor (CRF) secretion from the hypothalamus, thereby causing the anterior pituitary to stimulate adrenocorticotropic hormone (ACTH) synthesis and secretion. ACTH then activates the adrenal cortex to release glucocorticoids, directly affecting their peripheral target tissues, including the dermis of skin [46,47,48].

CREB is a well-characterized transcription factor, whose activity is regulated in various cell types by phosphorylation at Ser133. Genome-wide studies indicate that CREB binds to CRE, a target sequence that exists in various gene promoters and extensively regulates up to 5000 putative target genes [49,50]. It is worth noting that the binding ability of CREB to a specific target sequence is different among the cell types; thus, the CREB target gene family is different among cell types [51,52,53,54]. For example, forskolin, a cAMP activator, exposure reliably induced several target genes in embryonic kidney cells, as well as primary cultures of hepatocytes or pancreatic cells by gene profiling assays, but the sets of cAMP responsive genes in each case were almost completely different [51]. In our study using dermal fibroblasts, among the putative 34 and 12 CREB target genes related to cell survival and collagen synthesis, respectively, we found that OR51B5 knockdown selectively reduced the CREB target genes, including *RPS6*, *TFRC*, *TPT1*, *CREB3L1*, *CTGF*, and *RGCC*.

In the present study, the cell viability was markedly decreased in *OR51B5*-knockdown cells compared with control cells. Given that the cell viability can be affected not only by cell death, but also by the cell cycle, we cannot exclude the possibility that OR51B5 also contributes to the regulation of cell cycle in dermal fibroblasts; this needs to be explored in future studies.

## 4. Materials and Methods

### 4.1. Cell Culture

The human dermal fibroblast cell line (Hs68) was purchased from American Type Culture Collection (ATCC; Rockville, MD, USA). To the best of knowledge, Hs68 cell is currently the most widely used dermal fibroblast cell line for studying physiological processes in dermis, displaying typical characteristics of primary dermal fibroblasts; it is able to regulate various ECM components, including procollagen, in response to a variety of environmental stimuli (e.g., UV and stress hormone) [13,55,56,57]. The cells were grown in Dulbecco’s modified Eagle’s medium (DMEM; HyClone; Waltham, MA, USA) supplemented with 10% (*v*/*v*) fetal bovine serum (FBS, HyClone) and 1% penicillin–streptomycin (Gibco; Grand Island, NE, USA) at 37 °C in a 5% carbon dioxide atmosphere. The cells used in the experiments were between passages 5 and 10.

### 4.2. Cell Treatment

For developing experimental models of skin aging, Hs68 cells were seeded in 24-well plates and were incubated in a culture medium without FBS for 24 h. Then, the cells were washed with phosphate buffer saline (PBS; WelGENE; Daegu, Korea), stimulated with 20 mJ/cm^2^ UVB using a CL-1000 UV crosslinker (UVP; Cambridge, UK) or 1 μM dexamethasone (Sigma-Aldrich; Seoul, Korea), and further incubated in the same medium for another 24 h.

### 4.3. WST-1 Assay

Hs68 cells were cultured in 96-well plates for 24 h and incubated with the water-soluble tetrazolium salt-1 (WST-1, Sigma-Aldrich) diluted 1:10 in culture media for 2 h. Absorbance was determined immediately using an Infinite M200 microplate reader (Tecan; Groedig, Austria) at 450 nm against a blank without cells but containing culture medium and WST-1.

### 4.4. LDH Assay

LDH release into the culture supernatant was measured using an EZ-LDH assay kit (DoGenBio; Seoul, Korea), following the manufacturer’s instructions. Briefly, cells were cultured and treated as mentioned in Section 4.2. After that, the supernatant medium (10 μL) for each sample was transferred into a 96-well plate and was allowed to incubate with the LDH reaction mixture (100 μL) at 25 °C for 15 min in a dark room. LDH levels in the culture medium were determined by measuring the absorbance (450 nm) on an Infinite M200 microplate reader (Tecan).

### 4.5. Senescence-Associated β-Galactosidase (SA-β-gal) Staining

SA-β-gal staining was performed using a senescence beta-galactosidase cell staining kit (Cell Signaling Technology, MA, USA), following the manufacturer’s protocol. In brief, Hs68 cells were cultured in six-well plates and treated as mentioned in Section 4.2. After that, the cells were washed with PBS and fixed with a fixative solution for 10 min at 25 °C. The cells were then rinsed with PBS and incubated with a fresh β-gal staining solution at 37 °C overnight. The images were captured from five random fields per sample using a microscope (Nikon Eclipse TS2; Nikon, Tokyo, Japan) equipped with the DMX1200 camera (Nikon). The results are expressed as the percentage of SA-β-gal-positive cells in the total cell number.

### 4.6. Procollagen Type I C-Peptide Measurement

Procollagen type I C-peptide (PIP) in the culture supernatant collected from the cells was quantified using a PIP enzyme-linked immunosorbent assay (ELISA; MK101; Takara; Shiga, Japan), following the manufacturer’s instructions. The amount of PIP in each sample was normalized to the total cellular protein content measured by the Bradford assay (BioRad; Hercules, CA, USA).

### 4.7. RNA Extraction, cDNA Synthesis, and Polymerase Chain Reaction (PCR)

The total RNA from the cells was isolated using TRIzol reagent (Invitrogen; Carlsbad, CA, USA) following the manufacturer’s instructions. Briefly, 1 µg total RNA was used to generate cDNA using oligo(dT) primer and SuperScript IV reverse transcriptase (Invitrogen). Quantitative PCR was performed with cDNA, primers listed in Supplementary Table S1, and SYBR green (BioRad) on a CFX Real-Time System (BioRad) to measure the OR, cell survival-, and collagen biosynthesis-related gene expression. Glyceraldehyde 3-phosphate dehydrogenase (*GAPDH*) was used as the reference gene. The relative gene expression was quantified by the comparative “ct” (2^−ΔΔct^) method. Semi-quantitative PCR was performed using a 2× PCR Master Mix (Intron; Seoul, Korea) and primers listed in Supplementary Table S1 on a GeneMax thermal cycler (BIOER; Hanqzhou, China) to detect the olfactory signaling pathway components in Hs68 cells. The PCR products were separated by electrophoresis on 1.2% agarose gels containing TopRed Nucleic Acid Gel Stain (Biopure; Horndean, UK) and were photographed under UV light using a CoreBio i-MAX Gel Image Analysis System (CoreBio system; Seoul, Korea).

### 4.8. siRNA Screening

siRNAs oligonucleotides targeting specific olfactory receptors and non-targeting control siRNA were designed and purchased from Bioneer (Daejeon, Korea; Supplementary Table S2). Hs68 cells were silenced with the siRNA oligonucleotides (100 nM) or non-targeting siRNAs using the transfection reagent Lipofectamine 3000 (Invitrogen), according to the manufacturer’s protocol. siRNA-transfected cells and their supernatants were collected after incubating for 72 h for further experiments.

### 4.9. cAMP Measurement

Hs68 cells were washed in ice-cold PBS, harvested by centrifugation at 1800× *g* for 5 min, and extracted with 0.1 M HCl. The extracts were centrifuged at 15,000× *g* for 3 min and the supernatants were collected. The cAMP levels in the supernatants were measured using a direct cAMP ELISA kit (Enzo, Lausen Switzerland), according to the manufacturer’s instructions. cAMP values were individually normalized to the total protein concentrations measured by the Bradford assay for each sample.

### 4.10. Western Blot

Hs68 cells were lysed in a protein extraction buffer (iNtRON; Seoul, Korea). Proteins were separated by sodium dodecyl sulfate–polyacrylamide gel electrophoresis (SDS–PAGE) and transferred to a nitrocellulose membrane (Whatman; Kent, UK). The membrane was blocked for 1 h in 5% bovine serum albumin (BSA; MP Biomedicals; Auckland, New Zealand) in Tris-buffered saline/Tween-20 (TBST), and probed with primary antibodies at 4 °C overnight. The primary antibodies used were anti-PKA Cα (Cell Signaling; Herts, UK; 1:1000), anti-GAPDH (Cell Signaling; 1:5000), anti-pCREB (Cell Signaling; 1:1000), and anti-CREB (Cell Signaling; 1:1000). Then, the membrane was incubated with peroxidase-conjugated secondary antibody (Sigma) at 20 °C for 1 h. Protein levels were detected using an electrochemiluminescence detection reagent (ECL, BioRad) using Ez-capture (ATTO; Tokyo, Japan). Quantification was performed using the ImageJ program (National Institutes of Health; Bethesda, MD, USA).

### 4.11. Statistical Analysis

The results are expressed as mean ± standard error of mean (SEM). Statistical differences were measured using Student’s *t*-test using SPSS Statistics 26 (IBM; Armonk, NY, USA). In all of the analyses, statistical significance was set at *p* < 0.05.

## 5. Conclusions

In brief, we have demonstrated, for the first time, that OR51B5 knockdown can decrease cell survival and collagen synthesis in human dermal fibroblasts. OR51B5 knockdown also decreased cAMP levels and dampened the downstream PKA/CREB protein pathway, downregulating the survival and collagen synthesis-related genes in the dermal fibroblasts. Therefore, OR51B5 may be an interesting candidate that plays a role in cell survival and collagen synthesis, which warrants further investigation.

## Data Availability

All data generated or analyzed during this study are included in this published article.

## Supplementary Materials

The following are available online at https://www.mdpi.com/article/10.3390/ijms22179273/s1.
