# Supplementary material for: Dermal Olfactory Receptor OR51B5 Is Essential for Survival and Collagen Synthesis in Human Dermal Fibroblast (Hs68 Cells)
Source: Int J Mol Sci. 2021 Aug 27;22(17):9273. doi: 10.3390/ijms22179273 (PMC8431403; doi:10.3390/ijms22179273)
Supplement: Supplementary file 1 [file ijms-22-09273-s001.zip › ijms-1357350-supplementary.pdf]

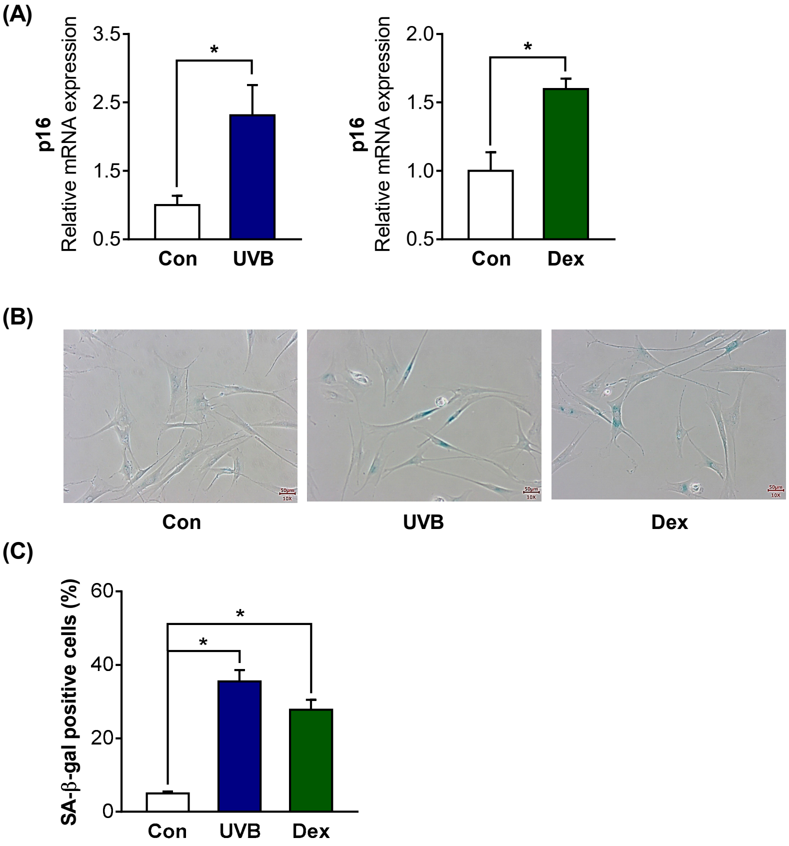

Figure S1. The effects of ultraviolet B (UVB) radiation or dexamethasone (Dex) on the cellular senescence markers in Hs68 cells. The cells were treated with or without 20 mJ/cm^2 UVB or 1 μM Dex. After treatment for 12 h, (**A**) the p16 mRNA levels were analyzed by quantitative polymerase chain reaction (PCR). Glyceraldehyde 3-phosphate dehydrogenase (*GAPDH*) was used as reference gene for PCR. (**B**) The cells were stained with the Senescence-associated β-galactosidase (SA-β-gal) staining kit. Representative image of SA-β-gal staining. (**C**) Percentage of SA-β-gal-positive cells. The results are expressed as mean ± standard error of mean (SEM) of three independent experiments. *p<0.05.

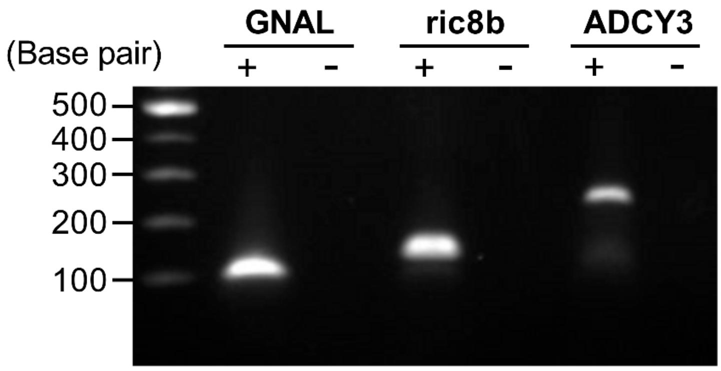

Figure S2. The presence of olfactory signaling pathway components in Hs68 cells. Agarose gel electrophoresis shows (amplification) semi-quantitative polymerase chain reaction (PCR) of olfactory signaling pathway components genes, including G Protein Subunit Alpha L (*GNAL*), *ric8b*, and adenylate cyclase type 3 (*ADCY3*) in Hs68 cells. +, reaction containing reverse transcriptase; −, reaction without reverse transcriptase.

Table S1. Sequences of primers used in this study.

Gene name	Primer sequences (5'→ 3')
p16	F: TGCCCAACGCACCGAATAG<br>R: CCCCTGCAAACTTCGTCCTC
Collagen type I alpha 1 chain (*COL1A1*)	F: TTGCTCCCCAGCTGTCTTAT<br>R: AGACCACGAGGACCAGAGG
Olfactory Receptor Family 1 Subfamily C Member 1 (*OR1C1*)	F: CACGCCCCTTGTCTGTCTGTATCC<br>R: TGGCTGTGCCGTAAAACAAC
OR2AE1	F: TGTGGCAGAAGAATCAGACC<br>R: GAGAATGGTGAGGGTGTTGC
OR2AG2	F: TCTACAGCCTGAGGAATAAGGA<br>R: GGAGGAATGGTGAGAGGCAA
OR2AK2	F: TCTATGCAACCACTCTCTTTACC<br>R: AAATACTGCCACCGCCTTATCC
OR2A1/42	F: GCATGACGCAGACCTTTCTCT<br>R: GCAGTCTTAGGATGAGAACCA
OR2A4/7	F: GACCTCATTCAACCTCACAATC<br>R: ATTTCATTCAGCGGCAAACAC
OR2B6	F: GTTGTCTACCCTGACTCTCCA<br>R: GCCTCATTTGCTGTTGTCTCA
OR2W3	F: ATGGATGGAACCAATGGCAG<br>R: GAGGTACGCGATCAGGATGA
OR7D2	F: TCAGCTACATGGAAGCAGGAA<br>R: GGACGATGCAAGTGCTGAAA
OR10A4	F: CCCTTGCACTACCCAGTCATC<br>R: TCAGAGCCTCCAGTTCAAACA
OR10A5	F: CAAAGGACACGGGCCAAAC<br>R: CAGCAATGCGAGTATAGGAAC
OR10AD1	F: TCCGCAGAGACAACCAACATAG<br>R: GGTGAGAGGCACAAGTAGAGA
OR51B4	F: CTGCTAGACCAGAGGGAGATT<br>R: TGTTCATCACTCGGGAATTGGT
OR51B5	F: AGAAACCAAGAGCCACGACT<br>R: GTGAGGCAACAACTCAGCAC
OR51I1	F: CCATGCTCAACCCTATCATCTA<br>R: GGCCTGGGATTTATGGAAGAA
OR52D1	F: TCTCCCCCTTCATCTTCTTGCT<br>R: GAGCCACAGTTAGCCCATAGA
ATPase Family AAA Domain Containing 3A (*ATAD3A*)	F: GCTGTTGGGGTCTACTCAGC<br>R: AAGGGACTCAGCCAGGAACT
Aurora Kinase B (*AURKB*)	F: GGAGTGCTTTGCTATGAGCTGC

Gene name	Primer sequences (5'→ 3')
	R: GAGCAGTTTGGAGATGAGGTCC
Cell Division Cycle 73 (*CDC73*)	F: GCCTTCCATCTGAAGTATGATGA<br>R: TGTACCTGTCCAATGTTTCCC
Cyclin Dependent Kinase 1 (*CDK1*)	F: TACAGGTCAAGTGGTAGCCA<br>R: AGCACATCCTGAAGACTGACT
Chromodomain Helicase DNA Binding Protein 8 (*CHD8*)	F: GGTCACACAAGACCCCATTGA<br>R: AGGGACCAGTTCACTTGCTG
α-Enolase 1 (*ENO1*)	F: ACGAGACCCAGTGGCTAGAAG<br>R: TGGGCTGTGGGTTCTAAGG
Frataxin (*FXN*)	F: AAGCCATACACGTTTGAGGACTA<br>R: TTGGCGTCTGCTTGTTGATCA
Histone Deacetylase 3 (*HDAC3*)	F: TTGAAGATGCTGAACCATGC<br>R: TGGCCTGCTGTAGTTCTCCT
Heterogeneous Nuclear Ribonucleoprotein K (*HNRNPK*)	F: AGACCTGGAGACCGTTAC<br>R: ATAAGCCATCTGCCATTC
Heat Shock Transcription Factor 1 (*HSF1*)	F: GAACAGCTTCCACGTGTTCG<br>R: TGGCCATGTTGTTGTGCTTG
Heat Shock Protein Family D (Hsp60) Member 1 (*HSPD1*)	F: TAGCTGTTACAATGGGGCCA<br>R: GGCAACGTCCTGAACAAGTT
Lysine Demethylase 1A (*KDM1A*)	F: TGACCGGATGACTTCTCAAGA<br>R: GTTGGAGAGTAGCCTCAAATGTC
Mediator Complex Subunit 1 (*MED1*)	F: TGATCTTCCTGCGTGTTTCTTC<br>R: ACAACGGGATTCCTGTGCAA
Mechanistic Target Of Rapamycin Kinase (*MTOR*)	F: CGCTGTCATCCCTTTATCG<br>R: ATGCTCAAACACCTCCACC
MYC proto-oncogene, bHLH transcription factor (*MYC*)	F: GCGCCCATTAATACCCTTCTTTC<br>R: CTCTCAAACCCTCTCCCTTTCTC
N-Alpha-Acetyltransferase 15, NatA Auxiliary Subunit (*NAA15*)	F: GCTTTGCTGTGGGCTTTATC<br>R: ACAAGACTTCCCTGAACTTACC
Nucleoporin 62 (*NUP62*)	F: GCCTCGGCTGAAGAAAGAAG<br>R: CTCTGGCTCCCAAAGCAAATC
PCI Domain Containing 2 (*PCID2*)	F: ATGGTGTGGACACGTCAAAG<br>R: TCAGCACAACCAAAGTGGAAAG
3-Phosphoinositide Dependent Protein Kinase 1 (*PDPK1*)	F: GGCGGAAACCCTTGCCTAAC<br>R: TATCGCTGCCTCCAAACCTC
Prohibitin 2 (*PHB2*)	F: AGCAGGAACAGCACAGAAGA<br>R: CGGAGCTTGATATAGCCAGGAT
Polo Like Kinase 1 (*PLK1*)	F: CCAGAGGGAGAAGATGTCCA<br>R: CAACACCACGAACACGAAGT
Protein Kinase, DNA-Activated, Catalytic Subunit (*PRKDC*)	F: GAACACCATGTCCCAAGAGGAG<br>R: GGCTTGTACTCATCCTCACG
Protein Tyrosine Phosphatase Non-Receptor Type 1 (*PTPN1*)	F: GCTGATACCTGCCTCTTGCTGATG<br>R: AGCTGGTCGGCTGTCTGGATC
Ribosomal Protein L10 (*RPL10*)	F: GATGCCAAGATTCGCATTTTTG

Gene name	Primer sequences (5'→ 3')
	R: AGCTGCTCATATTCATCTGACA
Ribosomal Protein S27a (*RPS27A*)	F: CTGGAAGATGGACGTACTTTGTC<br>R: CGACGAAGGCGACTAATTTGC
Ribosomal Protein S3A (*RPS3A*)	F: AAATCATGACCCGAGAGGT G<br>R: T TGGGCTTCTTCAGCAT TTT
Ribosomal Protein S6 (*RPS6*)	F: CTTCAGAGCAATACGCCGCCG<br>R: GCCGCATCCACACAGTCCGT
Splicing Factor Proline And Glutamine Rich (*SFPQ*)	F: CTGGGAAATGCATCTGGAGG<br>R: GCGTGCTTCCTATATGCCAA
SON DNA And RNA Binding Protein (*SON*)	F: TGGCCCTGATCATCGCAAAC<br>R: ACATACAATTGGGTCTGGTAAGG
Signal Transducer And Activator Of Transcription 5B (*STAT5B*)	F: CAGCCTGGACGTGCTACAGT<br>R: TGGTCACCAGGGCTGAGATA
Suv3 Like RNA Helicase (*SUPV3L1*)	F: GTTGAATTTGGAGGGCTTTCC<br>R: GCTGGGTGGCTCAGTAGCTT
Transferrin Receptor (*TFRC*)	F: GTCGCTGGTCAGTTCGTGATT<br>R: AGCAGTTGGCTGTTGTACCTCTC
Tumor Protein, Translationally-Controlled 1 (*TPT1*)	F: TATTGGACTACCGTGAGG<br>R: CAAGCAGAAGCCAGTTATG
TP53 Regulated Inhibitor Of Apoptosis 1 (*TRIAP1*)	F: GTGGCCTGGATTAGCAAAGAG<br>R: GGTTCTTCCTCCACAGCTATTC
Arrestin Beta 2 (*ARRB2*)	F: CCCTCAATGTAAATGTCCACG<br>R: GATGTCGGCGTACTGTCTCAC
Bone Morphogenetic Protein 4 (*BMP4*)	F: CCGCAGCCTAGCAAGAGTG<br>R: GCTCAGGATACTCAAGACCAGTG
Chromobox 8 (*CBX8*)	F: GTGTGTGTATGTGTCTCCTTCTC<br>R: TCACGACCGCAGAAGATAGG
cAMP Responsive Element Binding Protein 3 Like 1 (*CREB3L1*)	F: GAAGGTGGAGACCCTGGAGAATG<br>R: GAGCCCAGCACCAGAACAAAG
Connective tissue growth factor (*CTGF*)	F: CTGGGAAGCCATCACTTACCTTGC<br>R: GTTTCTAGAGTCGCTGGGAAGCTG
Endoglin (*ENG*)	F: AGCTGACTCTCCAGGCATCC<br>R: GCAGCTCTGTGGTGTTGACC
Coagulation Factor II Thrombin Receptor (*F2R*)	F: CCCGGGCTCAACATCACTAC<br>R: GCAACTGCGGAAGAGCTAAG
Histone Deacetylase 2 (*HDAC2*)	F: ATGGCGTACAGTCAAGGAGGC<br>R: AAATCAGAACAGCTCAGCAAC
Platelet Derived Growth Factor Receptor Beta (*PDGFRB*)	F: GCACTTTTATCCACCCAGGA<br>R: GTACTTGGCTCAGCCTCCAG
Regulator Of Cell Cycle (*RGCC*)	F: ACTTCCACTACGAGGAGCAC<br>R: TGAATCTGCACTCTCCGAGTC
RUNX Family Transcription Factor 1 (*RUNX1*)	F: TGAGCTGAGAAATGCTACCGC<br>R: ACTTCGACCGACAAACCTGAG
Transforming Growth Factor Beta 1 (*TGFB1*)	F: AACGCACTAACTTGACCTACAG

Gene name	Primer sequences (5'→ 3')
	R: CTCCAGAGTTCAAAGGCATCC
G Protein Subunit Alpha L (*GNAL*)	F: CAGCAAGACGACGGAAGACC<br>R: CGCTCTTTCTGCAACTGCTTCTC
Ric8b	F: TGTGACGGTAGACAGTTGGAAG<br>R: GCATTGCTGTGTAGCTCTTCTG
Adenylate Cyclase 3 (*ADCY3*)	F: GCCCAACTTTGCTGACTTCTAC<br>R: GCTCTCTCTCGGACTTGTCTTC
Glyceraldehyde 3-phosphate dehydrogenase (*GAPDH*)	F: ATCAAGAAGGTGGTGAAGCAG<br>R: GTCGCTGTTGAAGTCAGAGG

Table S2. Sequences of small interfering RNA (siRNA) oligonucleotides used in this study.

Gene name		siRNA sequence (Sense [S], antisense [A])
OR2AE1	#1	S: CAUUACGCCCACAUUGAAU<br>A: AUUCAAUGUGGGCGUAAUG
	#2	S: CCUCUAUUUGUGUCUAGGU<br>A: ACCUAGACACAAAUAGAGG
OR2AK2	#1	S: CUCUUCUCUUUGUCGUCAU<br>A: AUGACGACAAAGAGAAGAG
	#2	S: CACCAUUGUCACACCUCUA<br>A: UAGAGGUGUGACAAUGGUG
OR2A1/42	#1	S: CUGUCUAUGAUUGUCUCUA<br>A: UAGAGACAAUCAUAGACAG
	#2	S: CCUACCUGCGAAUCAUGGU<br>A: ACCAUGAUUCGCAGGUAGG
OR2A4/7	#1	S: CAUCAUUGAGCGGUUCUUA<br>A: UAAGAACCGCUCAAUGAUG
	#2	S: GAGGAUGAACCUGUCGUGU<br>A: ACACGACAGGUUCAUCCUC
OR10A4	#1	S: CUGAUCAUUCAAGACACAA<br>A: UUGUGUCUUGAAUGAUCAG
	#2	S: CUCUCUCUUCUAUAGCACU<br>A: AGUGCUAUAGAAGAGAGAG
OR10A5	#1	S: GAGCAAGAAGUUGUUAUCA<br>A: UGAUAACAACUUCUUGCUC
	#2	S: CUGAGAGCAAGAAGUUGUU<br>A: AACAACUUCUUGCUCUCAG
OR51B4	#1	S: CAUUCAUUCACUGGCCAUU<br>A: AAUGGCCAGUGAAUGAAUG
	#2	S: CUCAGAGGCAGUUCACUAC<br>A: GUAGUGAACUGCCUCUGAG
OR51B5	#1	S: GUAUUGUCACUCCCAUGUU<br>A: AACAUGGGAGUGACAAUAC
	#2	S: CGAGUCUGGCAUUCUGCUU<br>A: AAGCAGAAUGCCAGACUCG

OR51I1	#1	S: CUCAACUUUCAUCCUGCUU<br>A: AAGCAGGAUGAAAGUUGAG
	#2	S: UCAACACCUGCAUGUCACA<br>A: UGUGACAUGCAGGUGUUGA
OR52D1	#1	S: AGAUUCCAACCUCAGUGAU<br>A: AUCACUGAGGUUGGAAUCU
	#2	S: UCUAUGGAGCUAGAACCAA<br>A: UUGGUUCUAGCUCCAUAGA
Non-targeting siRNA	S: GAACUGAUGACAGGGAGGC<br>A: GCCUCCCUGUCAUCAGUUC	
